# Association of early life factors with dyslipidemia in children and adolescents: The CASPIAN-V study

**DOI:** 10.34172/hpp.2020.53

**Published:** 2020-11-07

**Authors:** Bahareh Vard, Arefeh Adham, Roya Riahi, Golgis Karimi, Mohammad Esmail Motlagh, Ramin Heshmat, Mostafa Qorbani, Roya Kelishadi

**Affiliations:** ^1^Child Growth and Development Research Center, Research Institute for Primordial Prevention of Non-Communicable Disease, Isfahan University of Medical Sciences, Isfahan, Iran; ^2^Pediatrics Department, School of Medicine, Isfahan University of Medical Sciences, Isfahan, Iran; ^3^Department of Nutrition and Dietetics, Faculty of Medicine, University Putra Malaysia, 43400 Seri Kembangan, Selangor, Malaysia; ^4^Pediatrics Department, Ahvaz Jundishapur University of Medical Sciences, Ahvaz, Iran; ^5^Chronic Diseases Research Center, Endocrinology and Metabolism Population Sciences Institute, Tehran University of Medical Sciences, Tehran, Iran; ^6^Non-Communicable Diseases Research Center, Alborz University of Medical Sciences, Karaj, Iran

**Keywords:** Children, Dyslipidemia, Breastfeeding, Complementary feeding

## Abstract

**Background:** This study aimed to investigate the association between prenatal/infancy factors and lipid profile in children and adolescents.

**Methods:** This multicentric national study was conducted in 30 provinces in Iran. It comprised 4200 participants, aged 7-18 years, from the fifth survey of a national surveillance program. History regarding birth weight, as well as the type of consumed milk and food during infancy was obtained from parents. In addition to physical examinations, fasting blood samples were obtained to assess the lipid profile of these students.

**Results:** Data from 3844 participants were available (91.5% participation rate), 52.4 % of students were boys. Mean (SD) age of participants was 12.3(3.2) years. Consuming cow milk in the first two years significantly increased the risk of high triglycerides (TG) (odds ratio [OR]:2.77, 95% CI: 1.32-5.85, P: 0.01), elevated low-density lipoprotein (LDL) (P<0.05) and low high-density lipoprotein (HDL) (P <0.05). Students who had consumed commercially made food as complementary feeding were 93% more likely to have high LDL (OR: 1.93, 95% CI=1.19-3.13, P: 0.01) and 90% more likely to have high TG than students who had consumed homemade food (OR: 1.90, 95% CI: 1.15-3.12, P: 0.01). The aforementioned figures were not significantly associated with an elevated total cholesterol (TC) level.

**Conclusion:** Our findings revealed that the history of using human milk and home-made food as complementary feeding was associated with better lipid profile in childhood and early adolescence. Increasing public knowledge in this regard might be useful for encouragement of healthier life prevention of chronic diseases.

## Introduction

Lipid structures, such as triglycerides (TG), lipoproteins, cholesterols and phospholipids have different roles and functions in human body.^1^ Defects in absorption, accumulation and metabolism of lipids may lead to various lipid disorders.^2,3^

One of the main concerns about lipid metabolism is the risk of future cardiovascular disease (CVD) and atherosclerosis. Nowadays, the role of cholesterol is well known in the pathogenesis of atherosclerosis, as well as low-density lipoprotein cholesterol (LDL-C) from this lipid group which is known as the most important lipoprotein involved in this condition.^4^ This process begins with the accumulation of fatty acid streaks in the vascular wall, resulting in adverse health outcomes.^5^ Many of the lipid-related disorders start developing from an early age. Studies have shown that some CVD risk factors in adulthood can be tracked to childhood.^6^

Lipid profile test is a screening tool for analyzing measurable blood lipid structures that can help predict the presence of disorders associated with any abnormal lipid status such as dyslipidemia.^7^ It has been found that prenatal and postnatal dietary factors such as breastfeeding, obesity in pregnancy, maternal lipid status and various childhood diets may affect the child’s lipid profile.^8^ Determining the factors related to the lipid status of children can be of paramount importance for prevention of further disorders in later years of life.

The aim of this study was to investigate the relationship of the prenatal and infancy factors with lipid profile of a nationally representative sample from the population of Iranian children and adolescents.

## Materials and Methods

This cross-sectional nationwide study was conducted among school students in 30 provinces in Iran.

### Subjects

Data for this study were derived from the fifth survey of the school-based surveillance program entitled “Childhood and Adolescence Surveillance and Prevention of Adult Non-communicable Disease” (CASPIAN-V) formerly conducted in 2015. The aim and methods of the main survey have been described in details previously.^9^

Briefly, multistage stratified cluster sampling method with equal cluster size was used to select 7-18- year old students of both genders living in urban and rural areas across Iran. To collect the biochemical test samples, the number of 4200 students was calculated.

### Measurement tool

Students’ questionnaire was designed in accordance with the World Health Organization-Global School Health Survey (WHO-GSHS) questionnaire (translated to Persian). Moreover, questions about early-life factors were asked from parents. Validity and reliability of both English and Persian questionnaires has been previously confirmed.^10,11^

### Measurements 

Demographic information about students including age, sex, living area, and number of household members was collected by trained interviewers. Findings from physical examinations were recorded by trained health-care staff. Weight with light clothes and barefoot height were measured to the nearest 0.1 kg and 0.1 cm, respectively.^9^ Body mass index (BMI) was calculated by dividing weight (kg) by squared height (m^2^). BMI was categorized in accordance with the WHO growth charts.^12^

### Physical activity (PA) and leisure time screen time (ST)

PA classification was based on times per week that the child was physically active for at least 30 minutes. The level of PA was classified as low (<2 times/week), moderate (2-4 times/week) and high (>4 times/week).^13^ In order to measure the screen time (ST), number of hours per day that students spent in front of the television and/or videos, personal computer, or electronic games were asked, then cumulative spent time front screen (ST) were divided into two categories of low (<2 hours/day) and high (≥2 hours/day).^13^

Healthy and unhealthy dietary habits were evaluated by using principle component analysis on usage of healthy food (milk, fruits and vegetables) and unhealthy food products (sweets, salty foods, soft drinks, and fast foods). Two components from this method (healthy and unhealthy dietary habits), were divided into three categories: low, moderate and high healthy/unhealthy dietary habits.

### Blood sampling and Lipid profile

Eligible students, accompanied with one of their parents, were referred to the laboratory. Then, 6ml of venous blood sampling was collected, while students were to be fasting for 12 hours prior to our sampling. All collection tubes were centrifuged at 2500–3000 ×g for 10 minutes. All samples were sent to Isfahan Mahdieh laboratory in a cold box. TG, total cholesterol (TC), LDL-C and high-density lipoprotein-cholesterol (HDL-C), were measured enzymatically by Hitachi Autoanalyzer^14,15^ to determine lipid profile. Atherogenic index was calculated by dividing TG to HDL-C.^16^ Serum TGs ≥100 mg/dL, TC ≥ 200 mg/dL, LDL-C ≥110 mg/dL, HDL-C <40 mg/dL (except in boys 15–18 years: <45 mg/dL) and atherogenic index >2.0^16^ were considered as abnormal lipid profile.^17^

### Breastfeeding duration 

Breastfeeding duration was assessed by the question from parents “how many months did your child consume breast feeding in the first two years of life?” The answers were categorized into five categories of not breastfed (0 month), up to 6 months, 6-12 months, 12-18 months and 18-24 months.

### Birth weight 

For the statistical analysis, students’ birth weight (BW) (g) was asked from parents and then was categorized into three levels: low (<2500 g), normal (2500-4000 g) and high (>4000 g).

### Socio-economic status 

The socio-economic status (SES) of families was evaluated using principle component analysis method on questions about parents’ education level, parents’ employment, home ownership status (home owner/rental), type of school (public/private), car ownership and having a personal computer. This method summarized these factors in the main component of SES, which is categorized to low (the first tertile), moderate (the second tertile) and high SES (the highest tertile).

Other studied variables consisted of the type of complementary feeding at the first two years of life (always homemade food/always commercial food/often homemade food/often formula), type of milk consumed at first two years of life (breast milk/formula/cow milk/mixed), father’s and mother’s age at the child’s birth, father’s and mother’s preconception weight and consanguinity of parents.

### Statistical analysis

Results are presented as mean (SD) for continuous variables and frequency (%) for categorical variables.

In this study high TG, high TC, high LDL, low HDL and high atherogenic index were considered as binary outcome (yes/no). Comparison of demographic characteristics, mean of lipid profile indices and prevalence of abnormal lipid profile according to the gender were evaluated and compared between different prenatal/infancy dietary groups using *t* test and Chi-square test. The multiple logistic regression models were used to evaluate the association of prenatal factors and lactation period with lipid profile after adjustment for potential confounders. The odds ratio with 95% confidence interval (OR 95% CI) for all predictive variables was presented based on crude model (Model I), adjusted model for sex, age, living area, PA, ST, diet (Model II) and the model additionally adjusted for family size, mother education, father education and other independent variables in this study (Model III). All statistical analyses were performed by SPSS v. 18.0 (PASW Statistics for Windows, Chicago: SPSS, Inc.). For all analyses, type 1 error of 0.05 and type 2 error of 0.2 were considered.

## Results

The participation rate was 91.5% (n= 3843), 52.4 % of students were boys and 72.2 % lived in urban area. The demographic characteristics of study participants divided by their gender are presented in Table 1. The frequency of high PA, breast milk consumption and homemade food consumption as complementary feeding was significantly higher in boys than girls (*P* < 0.05). Duration of the breast feeding was neither associated with gender of the baby nor body weight.

Mean (95% CI) of lipid profile indices and prevalence of hyperlipidemia factors (95% CI) according to gender are presented in Table 2. Mean values of TC and LDL were significantly higher in girls than in boys (*P* < 0.05). The prevalence of elevated LDL was significantly higher in girls (29.7%) than boys (26.4%) (*P* = 0.03). Interestingly, prevalence of low HDL was significantly higher in boys (32.7%) than girls (26%) (*P* <0.001).

Tables 3 and 4 present the association of prenatal factors and lactation period characteristics with the risk of elevated TG, elevated TC and LDL, and low HDL-C derived from multiple logistic regression analysis. Students who had consumed cow milk in the two first years of life had significantly higher odds for elevated TG than student with breast milk consumption history (OR: 2.77, 95% CI: 1.32-5.85, *P* =0.01). The odds of elevated TG in students who had always consumed formula as complementary feeding was 90% higher than students who had consumed homemade food (OR: 1.90, 95% CI: 1.15-3.12, *P* : 0.01). There was no significant association between prenatal factors (i.e. type of complementary feeding, type of milk, father’s and mother’s age at child’s birth, pre-conception parental weights, consanguinity in parents and BW) and lactation period with odds of an elevation TC in 6 to18 years (Table 3).

Consuming cow milk in the first two years significantly increased the odds of high LDL and low HDL (*P* < 0.05). Students who consumed formula food as complementary feeding were 93% more likely to have high LDL than students consumed breast milk (OR: 1.93, 95% CI: 1.19-3.13, *P* : 0.01). The odds of high LDL in students with familial marriage of parents was 25% lower than others (OR: 0.75, 95% CI: 0.61-0.91, *P* < 0.001) (Table 4).

Table 5 presents the association of prenatal factors and lactation period with the risk of elevated atherogenic index using multiple logistic regression. Consuming cow milk in the first two years significantly increased the odds of high atherogenic index (OR: 2.31, 95% CI: 1.08-4.21, *P* = 0.03).

## Discussion

The findings of the present study showed that the risk of elevated TG, low HDL and high atherogenic index was significantly higher in students with the history of cow milk consumption in infancy than in the breastfed individuals. On the other hand, consumption of formula as complementary food was associated with a higher risk of elevated LDL and elevated TG compared to the breast milk or homemade complementary food. The association between the type of breast milk substitute and changes in various lipid profile indices has been investigated in some earlier studies. They demonstrated that breastfeeding was associated with high concentrations of TG and LDL in infancy followed by lower levels of LDL and TG in adolescence.^12^ Our results are consistent with the abovementioned studies. It is documented that infants who are formula-fed have higher cholesterol synthesis than infants who are breastfed. Evidence has not yet shown whether differences in cholesterol synthesis and metabolism during infancy period would persist beyond weaning in to adulthood. Further research is needed to clarify the differences between breast milk and formula such as galactose levels or higher phytosterol protein in formula which might affect lipid metabolism.^18^ The aforementioned studies also reported that breast milk substitutes are highly prevalent among families with high-SES living in developing countries, as opposed to results from Western countries. Moreover, in rural communities or more underprivileged families who are less likely to be able to buy substitute resources, breastfeeding was observed to be more frequently used in female infants, so the budget could be preserved to obtain breast milk substitutes for other infants and the whole family.^19,20^ The difference between the lipid status of breast milk and milk substitutes depends on the type of the replacements, the type and amount of fatty acids present in the formula, and the diet of the mother, but the findings of the many related studies reported better lipid profile status in breastfed individuals compared to those consuming milk substitute.^21,22^ It is reported that breastfeeding in infancy might reduce the risk of none communicable diseases in older ages.^23^ Exclusive breastfeeding in early infancy may improve lipid profile in late adolescence. Moreover, it has positive affect on cardiovascular health, thus the use of breast milk should be encouraged.^18^

Furthermore, our results showed that children who were in families with low SES had a higher risk of low HDL than those in other families. This is consistent with some earlier findings.^24^ SES is affected by different factors such as living environment, educational level, health insurance coverage, lifestyle, mental stress, budget, quality of life, free time activities such as exercise and work. Naturally, improvement in these factors is associate with a reduction in the prevalence and severity of many disorders such as dyslipidemia.^25^

Moreover, our study indicated that male subjects had a more frequent history of breastfeeding, homemade food as complementary feeding, and higher physical activity categories compared to females. In addition, girls showed higher levels of TC and LDL while boys had lower HDL levels. Besides, the prevalence of high LDL and low HDL in girls was significantly higher than in boys. It seems that, female gender can be considered as a risk factor for dyslipidemia in this study. Association between genders and lipid profile status of children has been investigated in some previous studies. These studies showed that the mean TG and cholesterol levels were higher in girls compared to the boys and the prevalence of low HDL in boys was higher.^26^

There are some possible underlying mechanisms regarding the lipid profile status in relation to gender. Of these mechanisms, puberty is the one which should be taken into account.^27,28^ Changes in the lipid profile are observed during different stages of puberty, childhood and adolescence.^29^ For instance, changes in the expression of some genes, such as ABCA1 and apoA-1 are associated with a reduction in HDL-3 production and an alteration in esterified cholesterol concentrations.^30^ The difference in sex hormones, also, correlates with the difference in metabolism of lipids; for example, testosterone leads to an increase in HDL.^31^ Other factors such as environment, nutrition, individual habits, and physical activity also affect the lipid profile.^32^

One of the limitations in this study was the fact that we did not evaluate the physical activity level based on the BMI of the students for each year of the age. Another limitation of this study is that students’ lipid profile in this study was not evaluated based on the age of puberty or separated age years. As a result, further studies may yield more accurate results by considering the educational stages, ethnicity, and place of residence of the students. Some strengths of this study which might overcome its limitations are large number of sample size, and nationwide coverage. These strengths increase the generalizability of our findings.

## Conclusion

It can be concluded that the history of using breast milk and homemade food as complementary feeding was associated with better lipid profile in childhood and adolescence.

Boys showed higher weight and risk for lower HDL levels compared to girls. Low family SES was significantly associated with a higher risk of having low HDL levels. Using breast milk as the main type of milk consumed throughout infancy and healthy homemade food as complementary feeding might secure the chances of healthier lipid profile indices in older stages of childhood and adolescence ,which in turn acts out as a protecting factor against non-communicable diseases such as CVD, hypertension and diabetes mellitus. In order to achieve such goals, physicians and pediatricians should encourage breast feeding and the avoidance of commercially made food and formula in infancy as much as possible. Public knowledge about the long-term benefits of healthy eating in early life should be increased.

## Acknowledgements

The author would like to thank the large team working with this nationwide project, as well as all students and families who participated in this research.

## Funding

Data of this study were obtained from a national surveillance program. the current sub-study was conducted as a thesis approved in Isfahan university of medical Sciences.

## Competing interests

None to declare.

## Ethical approval

The Research& Ethics committee of Isfahan University of Medical Sciences approved the study protocol (Project number: 194049). After explaining the aims of the study, written consent was obtained from parents and verbal assent from participants. The ethical approval ID for the current study was IR.MUI.MED.REC.1397.014.

## Authors’ contributions

BV contributed in the study design, drafting and editing the paper. AA contributed in the study conduct, drafting and editing the paper. RR contributed in the statistical analysis, drafting and editing the paper. GK contributed in the study concept, drafting and editing the paper. MEM contributed in the study conduct, drafting and editing the paper. RH contributed in the study concept, drafting and editing the paper. MQ contributed in the statistical analysis, drafting and editing the paper. RK contributed in the concept, design and conduct of the study as well as in drafting and editing the paper. All authors approved the final version of the paper for submission and accept the responsibility of the paper content.

**Table 1:** Characteristics of participants according to gender: The CASPIAN-V study

	**Girl**	**Boy**	**Total**	***P*** **value**
Age (y)	12.3 (3.2)	12.4 (3.1)	12.3 (3.2)	<0.001*
BMI (kg/m^2^)	18.5 (4.7)	18.5 (4.9)	18.5 (4.4)	0.56
Living area				0.76
Urban	1318 (72%)	1458 (72.4%)	2776 (72.2%)	
Rural	513 (28%)	555 (27.6%)	1068 (27.8%)	
Physical activity				0.001*
Low	626 (36.2%)	579 (30.9%)	1205 (33.5%)	
Moderate	568 (32.9%)	627 (33.5%)	1195 (33.2%)	
High	533 (30.9%)	666 (35.6%)	1199 (33.3%)	
Screen time				0.48
< 2 h	1549 (86.6%)	1677 (85.8%)	3226 (86.2%)	
≥ 2 h	240 (13.4%)	278 (14.2%)	518 (13.8%)	
Healthy dietary				0.73
Low	485 (33.0%)	561 (34%)	1046 (33.5%)	
Moderate	477 (32.4%)	539 (32.7%)	1016 (32.6%)	
High	508 (34.6%)	549 (33.3%)	1057 (33.9%)	
Unhealthy dietary				0.43
Low	482 (32.8%)	506 (30.7%)	988 (31.7%)	
Moderate	516 (35.1%)	590 (35.8%)	1106 (35.5%)	
High	472 (32.1%)	553 (33.5%)	1025 (32.9%)	
Fathers' hyperlipidemia history				0.23
yes	225 (12.4%)	223 (11.1%)	448 (11.7%)	
No	1589 (87.6%)	1778 (88.9%)	3367 (88.3%)	
Mothers' hyperlipidemia history				0.16
Yes	150 (8.3%)	141 (7.1%)	291(7.6%)	
No	1664 (91.7%)	1858 (92.9%)	3522 (92.4%)	
Socio-economic statues (SES)				0.01*
Low	629 (36.3%)	615 (31.8%)	1244 (33.9%)	
Moderate	538 (31.0%)	682 (35.3%)	1220 (33.3%)	
High	568 (32.7%)	637 (32.9%)	1205 (32.8%)	
Familial marriage of parents				0.99
No	975 (54.1%)	1083 (54.1%)	2058 (54.1%)	
Yes	827 (45.9%)	918 (45.9%)	1745 (45.9%)	
Type of milk consumed				0.03*
Breast milk	1494 (81.8%)	1636 (81.3%)	3130 (81.5%)	
Formula	109 (6.0%)	91 (4.5%)	200 (5.2%)	
Cow’s milk	11 (0.6%)	7 (0.3%)	18 (0.5%)	
Mixed	213 (11.7%)	279 (13.9%)	492 (12.8%)	
Type of complementary feeding				0.67
Always homemade food	1331 (73.6%)	1496 (74.5%)	2827 (74.1%)	
Always formula	63 (3.5%)	79 (3.9%)	142 (3.7%)	
Usually homemade food	362 (20.0%)	374 (18.6%)	736 (19.3%)	
Usually formula	52 (2.9%)	58 (2.9%)	110 (2.9%)	
Breastfeeding duration (months)				0.06
No feeding	73 (4.0%)	59 (3.0%)	132 (3.5%)	
To 6	499 (27.6%)	611 (30.6%)	1110 (29.2%)	
>6-12	167 (9.2%)	208 (10.4%)	375 (9.9%)	
>12-18	246 (13.6%)	252 (12.6%)	498 (13.1%)	
>18-24	821 (45.5%)	870 (43.5%)	1691 (44.4%)	
Birth weight (g)				0.94
< 2500	177 (10.8%)	195 (10.5%)	372 (10.7%)	
2500-4000	1334 (81.4%)	1517 (81.9%)	2851 (81.7%)	
>4000	127 (7.8%)	140 (7.6%)	267 (7.7%)	

* P < 0.05 considered as statistically significant.

**Table 2:** Mean (95% CI) of and prevalence (95% CI) of lipid profile in gender category: The CASPIAN-V study

	**Girls**	**Boys**	**Total**	***P*** **value**
**Mean (95% CI)**				
TG (mg/dL)	89.02 (87.04-91.11)	87.15 (85.24-89.18)	88.04 (86.56-89.50)	0.21
TC (mg/dL)	154.82(153.56-156.09)	152.96 (151.71-154.23)	153.85 (152.98- 154.73)	0.03*
LDL (mg/dL)	97.0 (95.25-98.82)	94.39 (92.72-96.23)	95.63 (94.48-96.89)	0.04*
HDL (mg/dL)	46.16 (45.71-46.61)	46.21 (45.75-46.66)	46.19 (45.86-46.48)	0.86
**Prevalence (95% CI)**				
High TG	28.6% (26.5-30.6)	26.9% (25-28.9)	27.7% (26.2-29.1)	0.29
High TC	4.9% (3.9-5.9)	5.0% (4.0- 6.0)	4.9% (4.3-5.6)	0.88
High LDL	29.7% (27.5-31.7)	26.4% (26.2-29.1)	28.0% (26.6-29.4)	0.03*
Low HDL	26.0% (23.9-28.0)	32.7% (30.8-34.8)	29.5% (28.0-31.0)	<0.001*

TG, triglyceride; TC, total cholesterol; LDL, low-density lipoprotein; HDL, high-density lipoprotein. * P < 0.05 considered as statistically significant.

**Table 3:** Association of prenatal factors and lactation period with lipid profile indices using logistic regression model: The CASPIAN-V study

	**High TG**						**High Total cholesterol**					
	**OR** ^a^ **(95%CI)**	***P*** **value**	**OR** ^b^ **(95%CI)**	***P*** **value**	**OR** ^c^ **(95%CI)**	***P*** **value**	**OR** ^a^ **(95%CI)**	***P*** **value**	**OR** ^b^ **(95%CI)**	***P*** **value**	**OR** ^c^ **(95%CI)**	***P*** **value**
**SES**												
Low	1.08 (0.91 1.29)	0.38	0.95 (0.79 1.15)	0.62	1.18(0.91 1.51)	0.21	0.78 (0.54 1.13)	0.19	0.70 (0.47 1.05)	0.08	0.74 (0.44 1.24)	0.25
Moderate	1.10 (0.92 1.31)	0.30	1.08 (0.90 1.31)	0.40	1.10(0.87 1.40)	0.43	0.97 (0.68 1.38)	0.87	0.92 (0.63 1.34)	0.65	1.00 (0.64 1.58)	1.00
High	1	-	1	-	1	-	1	-	1	-	1	-
**Type of complementary feeding**												
Always homemade food	1	-	1	-	1	-	1	-	1	-	1	**-**
Always formula	1.31 (0.91 1.87)	0.14	1.60 (1.17 2.21)	<0.001*	1.90 (1.15 3.12)	0.01*	0.69 (0.28 1.70)	0.41	1.91 (0.85 4.30)	0.12	1.14 (0.44 2.95)	0.78
Usually homemade foods	1.06 (0.89 1.27)	0.51	0.97 (0.78 1.21)	0.78	1.23 (0.95 1.59)	0.11	0.77 (0.51 1.16)	0.21	0.75 (0.47 1.22)	0.25	0.81 (0.47 1.39)	0.52
Usually formula	0.82 (0.52 1.28)	0.38	0.69 (0.45 1.05)	0.08	0.94 (0.50 1.74)	0.84	1.88 (0.96 3.67)	0.07	0.82 (0.33 2.07)	0.68	2.03 (0.78 5.29)	0.15
**Type of milk consumed**												
Breast feeding	1	-	1	-	1	-	1	-	1	-	1	**-**
Formula	0.84 (0.61 1.18)	0.41	0.76 (0.51 1.14)	0.19	0.82 (0.45 1.50)	0.51	0.96 (0.50 1.86)	0.91	1.13 (0.54 2.37)	0.75	1.69 (0.60 4.74)	0.32
Cow’s milk	2.02 (1.23 3.34)	0.01*	2.45 (1.37 4.39)	<0.001*	2.77 (1.32 5.85)	0.01*	0.59 (0.14 2.44)	0.47	0.83 (0.20 3.47)	0.79	1.89 (0.52 6.82)	0.33
Mixed	0.94 (0.75 1.17)	0.32	0.99 (0.77 1.29)	0.96	1.04 (0.75 1.46)	0.80	0.64 (0.37 1.09)	0.10	0.70 (0.38 1.28)	0.25	0.97 (0.49 1.91)	0.92
**Father** ^'^ **s age at the child birth**												
≤27 years	0.96 (0.81 1.14)	0.65	0.92 (0.75 1.13)	0.43	1.08(0.79 1.48)	0.61	1.13 (0.79 1.62)	0.51	1.10 (0.73 1.67)	0.65	1.02 (0.56 1.85)	0.95
28-33 years	1.06 (0.89 1.27)	0.49	1.08 (0.88 1.33)	0.45	1.25(0.96 1.64)	0.10	1.09 (0.76 1.58)	0.63	1.13 (0.74 1.72)	0.58	0.97 (0.57 1.64)	0.90
≥ 34 years	1	-	1	-	1	-	1	-	1	-	1	-
**Mother** ^'^ **s age at the child birth**												
≤22 years	0.92 (0.76 1.10)	0.34	0.90 (0.73 1.10)	0.31	0.80(0.58 1.10)	0.17	0.97 (0.65 1.43)	0.86	1.15 (0.74 1.79)	0.54	0.94 (0.50 1.75)	0.84
23-28 years	1.05 (0.89 1.25)	0.55	1.03 (0.84 1.26)	0.76	0.94(0.72 1.24)	0.67	1.38 (0.96 1.97)	0.08	1.29 (0.84 1.98)	0.25	1.09 (0.64 1.87)	0.74
≥ 29 years	1	-	1	-	1	-	1	-	1	-	1	-
**Mother** ^'^ **s weight at wedding**												
≤ 49 kg	1.06 (0.89 1.26)	0.53	1.07 (0.86 1.34)	0.54	1.07 (0.81 1.41)	0.64	0.89 (0.62 1.27)	0.52	1.03 (0.63 1.68)	0.91	1.26 (0.71 2.24)	0.43
49-59 kg	1.04 (0.86 1.24)	0.71	1.00 (0.82 1.22)	1.00	1.00 (0.79 1.26)	0.97	0.85 (0.59 1.22)	0.38	1.37 (0.90 2.08)	0.14	1.59 (0.99 2.53)	0.05
>59 kg	1	-	1	-	1	-	1	-	1	-	1	-
**Father** ^'^ **s weight at wedding**													
≤ 60 kg	1.01 (0.85 1.19)	0.93	1.01 (0.83 1.24)	0.90	0.87 (0.68 1.11)	0.26	0.72 (0.52 1.00)	0.05	0.74 (0.50 1.10)	0.14	0.63 (0.39 1.01)	0.06
60-70 kg	1.07 (0.88 1.29)	0.52	1.10 (0.87 1.38)	0.42	1.13 (0.87 1.47)	0.35	0.52 (0.34 0.80)	<0.001*	0.68 (0.42 1.10)	0.12	0.66 (0.40 1.10)	0.11
>70 kg	1	-	1	-	1	-	1	-	1	-	1	-
**Family relationship**												
No	0.92 (0.80 1.06)	0.24	0.92 (0.78 1.09)	0.32	0.87 (0.71 1.07)	0.19	0.94 (0.70 1.26)	0.68	0.91 (0.65 1.28)	0.58	0.79 (0.54 1.16)	0.23
Yes	1 -	-	1	-	1	-	1	-	1	-	1	-
**Breastfeeding duration**												
	1	-	1	-	1	**-**	1	-	1	-	1	**-**
To 6 month	1.38 (0.91 2.11)	0.13	1.25 (0.77 2.02)	0.36	0.90 (0.42 1.95)	0.79	1.01 (0.43 2.40)	0.98	0.74 (0.30 1.79)	0.50	0.50 (0.15 1.72)	0.44
>6-12 month	0.87 (0.54 1.40)	0.56	0.71 (0.41 1.22)	0.21	050 (0.21 1.19)	0.12	1.12 (0.44 2.87)	0.81	0.68 (0.25 1.89)	0.46	0.62 (0.15 2.62)	0.82
>12-18 month	1.06 (0.62 1.82)	0.84	0.99 (0.54 1.83)	0.98	1.07 (0.42 2.73)	0.88	1.41 (0.50 3.99)	0.52	1.31 (0.45 3.76)	0.62	1.19 (0.27 5.19)	0.52
>18-24 month	1.28 (0.84 1.93)	0.25	1.18 (0.74 1.88)	0.50	0.94 (0.42 2.10)	0.89	1.09 (0.47 2.53)	0.84	0.75 (0.31 1.77)	0.51	0.60 (0.16 2.20)	0.27
**Birth weight**												
2500-4000 kg	1	-	1	-	1	-	1	-	1	-	1	-
<2500 kg	0.97 (0.76 1.24)	0.80	1.01 (0.77 1.33)	0.94	1.00(0.72 1.38)	0.99	1.08 (0.67 1.74)	0.77	1.21 (0.71 2.06)	0.49	1.52 (0.86 2.69)	0.15
>400kg	1.09 (0.83 1.43)	0.55	1.08 (0.79 1.49)	0.62	1.09(0.76 1.58)	0.63	0.66 (0.33 1.31)	0.24	0.40 (0.15 1.11)	0.08	0.58 (0.23 1.45)	0.25

**Table 4:** Association of prenatal factors and lactation period with lipid profile indices using logistic regression model: The CASPIAN-V study

	**High LDL**						**Low HDL**					
	**OR (95%CI)**	**P-value**	**OR** ^a^ **(95%CI)**	**P-value**	**OR** ^b^ **(95%CI)**	***P*** **value**	**OR (95%CI)**	***P*** **value**	**OR** ^a^ **(95%CI)**	***P *** **value**	**OR** ^b^ **(95%CI)**	***P *** **value**
**SES**												
Low	1.09 (0.91 1.30)	0.34	1.03 (0.84 1.27)	0.77	0.92(0.72 1.17)	0.47	1.15 (0.97 1.37)	0.11	1.20(0.98 1.47)	0.08	1.31 (1.02 1.68)	0.03*
Moderate	1.08 (0.91 1.29)	0.37	1.07 (0.87 1.31)	0.54	0.90(0.72 1.13)	0.38	1.11 (0.93 1.33)	0.23	1.08 (0.88 1.33)	0.45	1.11 (0.87 1.40)	0.40
High	1	-	1	-	1	-	1	-	1	-	1	-
**Type of complementary feeding**												
Always homemade food	1	-	1	-	1	-	1	-	1	-	1	**-**
Always formula	1.26 (0.88 1.08)	0.20	1.62 (1.11 2.39)	0.01*	1.93 (1.19 3.13)	0.01*	0.82 (0.56 1.20)	0.30	0.91 (0.60 1.39)	0.67	0.96 (0.57 1.61)	0.88
Usually homemade foods	0.98 (0.82 1.17)	0.83	0.95 (0.77 1.18)	0.66	1.15 (0.89 1.48)	0.29	0.93 (0.78 1.11)	0.41	0.98 (0.79 1.21)	0.85	0.88 (0.68 1.14)	0.34
Usually formula	0.68 (0.42 1.81)	0.10	0.67 (0.40 1.14)	0.14	0.81 (0.44 1.50)	0.51	0.95 (0.63 1.45)	0.82	0.96 (0.58 1.59)	0.88	0.66 (0.35 1.26)	0.21
**Type of milk consumed**												
Breast feeding	1	-	1	-	1	-	1	-	1	-	1	**-**
Formula	1.14 (0.83 1.55)	0.41	1.09 (0.75 1.42)	0.47	1.38 (0.79 2.42)	0.51	0.79 (0.57 1.10)	0.16	0.73 (0.49 1.09)	0.12	0.78 (0.42 1.45)	0.43
Cow’s milk	1.56 (0.93 2.60)	0.09	1.42 (0.77 2.62)	0.26	2.49 (1.49 5.80)	<0.001*	1.33 (0.79 2.22)	0.28	1.48 (0.81 2.71)	0.21	2.16 (1.08 4.31)	0.03*
Mixed	0.93 (0.75 1.17)	0.55	1.10 (0.85 1.59)	0.64	1.35 (0.98 1.87)	0.07	0.97 (0.78 1.21)	0.81	1.06 (0.82 1.37)	0.64	1.30 (0.95 1.78)	0.19
**Father** ^'^ **s age at the child birth**												
≤27 years	0.93 (0.78 1.11)	0.44	0.83 (0.68 1.02)	0.07	0.83 (0.61 1.13)	0.24	0.97 (0.82 1.15)	0.75	0.97 (0.80 1.19)	0.78	1.10 (0.81 1.50)	0.54
28-33 years	1.04 (0.88 1.24)	0.64	1.00 (0.82 1.23)	0.98	1.06 (0.82 1.38)	0.64	0.98 (0.83 1.17)	0.86	0.94 (0.77 1.16)	0.58	0.98 (0.75 1.27)	0.85
≥ 34 years	1	-	1	-	1	-	1	-	1	-	1	-
**Mother** ^'^ **s age at the child birth**											
≤22 years	0.93 (0.78 1.12)	0.44	0.90 (0.73 1.11)	0.33	0.95 (0.69 1.31)	0.77	0.98 (0.83 1.17)	0.85	0.99 (0.81 1.21)	0.91	0.87 (0.64 1.20)	0.41
23-28 years	1.12 (0.94 1.33)	0.20	1.08 (0.88 1.32)	0.44	1.09 (0.83 1.42)	0.54	0.92 (0.78 1.10)	0.37	0.91 (0.74 1.11)	0.36	0.87 (0.67 1.14)	0.33
≥ 29 years	1	-	1	-	1	-	1	-	1	-	1	-
**Mother** ^'^ **s weight at wedding**									
≤ 49 kg	1.09 (0.91 1.30)	0.33	1.10 (0.90 1.36)	0.36	1.16 (0.89 1.53)	0.27	1.00 (0.83 1.21)	0.96	1.08 (0.87 1.34)	0.49	0.92 (0.70 1.21)	0.56
49-59 kg	1.12 (0.94 1.34)	0.21	1.14 (0.92 1.40)	0.23	1.05 (0.83 1.32)	0.70	0.87 (0.74 1.03)	0.11	0.95 (0.78 1.15)	0.58	0.89 (0.71 1.12)	0.32
>59 kg	1	-	1	-	1	-	1	-	1	-	1	-
**Father** ^'^ **s weight at wedding**									
≤ 60 kg	0.94 (0.79 1.12)	0.49	0.93 (0.76 1.13)	0.46	0.86 (0.67 1.10)	0.24	1.05 (0.89 1.24)	0.58	1.04 (0.86 1.26)	0.69	1.03 (0.81 1.31)	0.81
60-70 kg	1.05 (0.87 1.28)	0.60	1.05 (0.84 1.32)	0.66	1.06 (0.82 1.37)	0.67	0.93 (0.77 1.13)	0.49	0.94 (0.75 1.18)	0.59	0.95 (0.73 1.23)	0.69
>70 kg	1	-	1	-	1	-	1	-	1	-	1	-
**Family relationship**												
No	0. 89 (0.77 1.02)	0.10	0.82 (0.70 0.95)	0.01*	0.75 (0.61 0.91)	<0.001*	0.94 (0.82 1.08)	0.39	0.95 (0.81 1.12)	0.57	0.99 (0.82 1.21)	0.95
Yes	1 -	-	1	-	1	-	1	-	1	-	1	-
**Breastfeeding duration**												
	1	-	1	-	1	-	1	-	1	-	1	**-**
To 6 month	0.90 (0.61 1.33)	0.59	0.94 (0.60 1.47)	0.77	0.72 (0.36 1.58)	0.36	1.02 (0.69 1.52)	0.91	0.93 (0.59 1.48)	0.76	1.08 (0.50 2.35)	0.80
>6-12 month	0.66 (0.42 1.02)	0.06	0.56 (0.33 0.94)	0.03*	0.50 (0.22 2.82)	0.09	0.98 (0.63 1.51)	0.92	0.92 (0.55 1.53)	0.74	0.93 (0.39 2.20)	0.95
>12-18 month	0.77 (0.46 1.28)	0.31	0.83 (0.46 1.48)	0.52	1.18 (0.49 1.13)	0.71	0.86 (0.51 1.43)	0.55	0.90 (0.50 1.63)	0.73	1.03 (0.40 2.69)	0.87
>18-24 month	0.82 (0.56 1.20)	0.31	0.83 (0.53 1.29)	0.40	0.76 (0.36 1.45)	0.46	1.01 (0.68 1.48)	0.98	0.97 (0.62 1.51)	0.88	1.11 (0.50 2.49)	0.84
**Birth weight**												
2500-4000 kg	1	-	1	-	1	-	1	-	1	-	1	**-**
<2500 kg	0.95 (0.74 1.21)	0.67	1.02 (0.77 1.34)	0.89	1.16 (0.85 1.57)	0.36	0.89 (0.70 1.13)	0.33	0.77 (0.58 1.01)	0.06	0.78 (0.56 1.08)	0.14
>400kg	1.10 (0.84 1.45)	0.48	1.11 (0.81 1.52)	0.53	1.19 (0.84 1.70)	0.33	0.92 (0.69 1.22)	0.56	0.92 (0.67 1.27)	0.62	1.17 (0.83 1.66)	0.37

**Table 5:** Association of prenatal factors and lactation period with atherogenic index using logistic regression model: The CASPIAN-V study

	**High atherogenic index (TG/HDL-C > 2.0)**					
	**Model I**		**Model II**		**Model III**	
	**OR (95% CI)**	***P*** **value**	**OR (95%CI)**	***P*** **value**	**OR (95%CI)**	***P*** **value**
Type of complementary feeding						
Always homemade food	1	-	1	-	1	-
Always formula	1.22 (0.84-1.77)	0.31	1.57 (1.05-2.35)	0.03*	1.57 (0.98-2.52)	0.06
Usually homemade foods	1.10 (0.92-1.33)	0.30	1.10 (0.88 -1.37)	0.39	1.12 (0.88-1.42)	0.35
Usually formula	0.87 (0.55-1.37)	0.54	0.91 (0.53-1.56)	0.74	0.97 (0.56-1.67)	0.90
Type of milk consumed						
Breast milk	1	-	1	-	1	-
Formula	0.82 (0.58-1.16)	0.26	0.78 (0.51-1.19)	0.25	1.42 (0.83-1.53)	0.20
Cow’s milk	2.04 (1.23-3.39)	0.01	2.32 (1.29-4.18)	0.01*	2.13 (1.08-4.21)	0.03*
Mixed	0.81 (0.64-1.03)	0.08	0.85 (0.64-1.13)	0.26	1.13 (0.84-2.44)	0.43
Father^'^s age at the child birth (y)						
≤ 27	0.95 (0.79-1.14)	0.60	0.86 (0.70-1.06)	0.15	1.01 (0.76-1.35)	0.92
28-33	0.99 (0.83-1.19)	0.93	0.93 (0.75-1.15)	0.50	1.05 (0.82-1.33)	0.72
≥ 34	1	-	1	-	1	-
Mother^'^s age at the child birth (y)						
≤ 22	0.98 (0.81-1.18)	0.84	0.90 (0.72-1.11)	0.33	0.98 (0.73-1.31)	0.89
23-28	1.13 (0.94-1.35)	0.19	1.07 (0.86-1.31)	0.55	1.00 (0.78-1.28)	0.98
≥ 29	1	-	1	-	1	-
Pre-conception mother^'^s weight (kg)						
≤ 49	1.05 (0.86-1.27)	0.66	1.13 (0.90-1.42)	0.27	1.03 (0.81-1.33)	0.79
49-59	0.90 (0.75-1.07)	0.23	0.87 (0.70-1.07)	0.18	0.89 (0.72-1.10)	0.27
>59	1	-	1	-	1	-
Pre-conception father^'^s weight (kg)						
<60	1.01 (0.84-1.20)	0.95	1.09 (0.89-1.34)	0.41	0.95 (0.76-1.19)	0.64
60-70	1.10 (0.90-1.34)	0.36	1.20 (0.94-1.52)	0.14	1.13 (0.89-1.44)	0.30
>70	1	-	1	-	1	-
Familial marriage of parents						
No	0. 88 (0.76-1.02)	0.09	0.87 (0.73-1.03)	0.10	1.00 (0.83-1.20)	1.00
Yes	1	-	1	-	1	-
Breastfeeding duration (month)						
No feeding	1	-	1	-	1	-
To 6	1.44 (0.92-2.25)	0.12	1.28 (0.77-2.12)	0.35	1.39 (0.69-2.80)	0.36
>6-12	0.95 (0.58-1.57)	0.85	0.90 (0.51-1.58)	0.70	1.14 (0.52-2.51)	0.74
>12-18	1.32 (0.76-2.32)	0.33	1.29 (0.69-2.43)	0.42	1.63 (0.69-3.86)	0.27
>18-24	1.38 (0.89-2.14)	0.16	1.26 (0.77-2.08)	0.36	1.62 (0.78-3.37)	0.20
Birth weight (kg)						
2500-4000	1	-	1	-	1	-
<2500	1.02 (0.79-1.31)	0.88	1.08 (0.82-1.44)	0.58	0.95 (0.71-1.28)	0.76
>4000	1.26 (0.95-1.66)	0.11	1.22 (0.89-1.69)	0.22	1.29 (0.93-1.78)	0.13

Model I: without adjust (crude OR). Model II: adjusted for sex, age, region, physical activity, screen time, diet. Model III: additionally adjusted for BMI, hyperlipidemia history, SES and all variables present in this table. *P < 0.05 considered as statistically significant.

## References

[1] Sanders FW, Griffin JL (2016). De novo lipogenesis in the liver in health and disease: more than just a shunting yard for glucose. Biol Rev Camb Philos Soc.

[2] Moradi H, Vaziri ND (2018). Molecular mechanisms of disorders of lipid metabolism in chronic kidney disease. Front Biosci (Landmark Ed).

[3] Thompson GR (1989). Lipid related consequences of intestinal malabsorption. Gut.

[4] Orozco-Beltran D, Gil-Guillen VF, Redon J, Martin-Moreno JM, Pallares-Carratala V, Navarro-Perez J (2018). Correction: Lipid profile, cardiovascular disease and mortality in a Mediterranean high-risk population: the ESCARVAL-RISK study. PLoS One.

[5] Dabas A, Yadav S, Gupta VK (2014). Lipid profile and correlation to cardiac risk factors and cardiovascular function in type 1 adolescent diabetics from a developing country. Int J Pediatr.

[6] Daniels SR, Greer FR (2008). Lipid screening and cardiovascular health in childhood. Pediatrics.

[7] Lozano P, Henrikson NB, Dunn J, Morrison CC, Nguyen M, Blasi PR (2016). Lipid screening in childhood and adolescence for detection of familial hypercholesterolemia: evidence report and systematic review for the US Preventive Services Task Force. JAMA.

[8] Scartezini M, dos Santos Ferreira CE, Izar MCO, Bertoluci M, Vencio S, Campana GA (2017). Positioning about the flexibility of fasting for lipid profiling. Arq Bras Cardiol.

[9] Motlagh ME, Ziaodini H, Qorbani M, Taheri M, Aminaei T, Goodarzi A (2017). Methodology and early findings of the fifth survey of childhood and adolescence surveillance and prevention of adult noncommunicable disease: the CASPIAN-V study. Int J Prev Med.

[10] Kelishadi R, Motlagh ME, Roomizadeh P, Abtahi SH, Qorbani M, Taslimi M (2013). First report on path analysis for cardiometabolic components in a nationally representative sample of pediatric population in the Middle East and North Africa (MENA): the CASPIAN-III study. Ann Nutr Metab.

[11] World Health Organization (WHO). Physical Status: The Use and Interpretation of Anthropometry. Geneva: WHO; 1995.

[12] WHO Child Growth Standards based on length/height, weight and age. Acta Paediatr Suppl 2006;450:76-85. doi: 10.1111/j.1651-2227.2006.tb02378.x. 16817681

[13] Gademan MG, Vermeulen M, Oostvogels AJ, Roseboom TJ, Visscher TL, van Eijsden M (2014). Maternal prepregancy BMI and lipid profile during early pregnancy are independently associated with offspring’s body composition at age 5-6 years: the ABCD study. PLoS One.

[14] McNamara JR, Schaefer EJ (1987). Automated enzymatic standardized lipid analyses for plasma and lipoprotein fractions. Clin Chim Acta.

[15] Friedewald WT, Levy RI, Fredrickson DS (1972). Estimation of the concentration of low-density lipoprotein cholesterol in plasma, without use of the preparative ultracentrifuge. Clin Chem.

[16] Yoo DY, Kang YS, Kwon EB, Yoo EG (2017). The triglyceride-to-high density lipoprotein cholesterol ratio in overweight Korean children and adolescents. Ann Pediatr Endocrinol Metab.

[17] Zimmet P, Alberti G, Kaufman F, Tajima N, Silink M, Arslanian S (2007). The metabolic syndrome in children and adolescents. Lancet.

[18] Hui LL, Kwok MK, Nelson EAS, Lee SL, Leung GM, Schooling CM. Breastfeeding in infancy and lipid profile in adolescence. Pediatrics 2019;143(5). 10.1542/peds.2018-3075 30967484

[19] Jain A, Tyagi P, Kaur P, Puliyel J, Sreenivas V (2014). Association of birth of girls with postnatal depression and exclusive breastfeeding: an observational study. BMJ Open.

[20] Fledderjohann J, Agrawal S, Vellakkal S, Basu S, Campbell O, Doyle P (2014). Do girls have a nutritional disadvantage compared with boys? statistical models of breastfeeding and food consumption inequalities among Indian siblings. PLoS One.

[21] Robinson S, Fall C (2012). Infant nutrition and later health: a review of current evidence. Nutrients.

[22] Martin CR, Ling PR, Blackburn GL. Review of infant feeding: key features of breast milk and infant formula. Nutrients 2016;8(5). 10.3390/nu8050279 PMC4882692 27187450

[23] Stuebe A (2009). The risks of not breastfeeding for mothers and infants. Rev Obstet Gynecol.

[24] Owen CG, Whincup PH, Odoki K, Gilg JA, Cook DG (2002). Infant feeding and blood cholesterol: a study in adolescents and a systematic review. Pediatrics.

[25] Singhal A, Cole TJ, Fewtrell M, Lucas A (2004). Breastmilk feeding and lipoprotein profile in adolescents born preterm: follow-up of a prospective randomised study. Lancet.

[26] Victora CG, Horta BL, Post P, Lima RC, De Leon Elizalde JW, Gerson BM (2006). Breast feeding and blood lipid concentrations in male Brazilian adolescents. J Epidemiol Community Health.

[27] Herting MM, Sowell ER (2017). Puberty and structural brain development in humans. Front Neuroendocrinol.

[28] Li W, Liu Q, Deng X, Chen Y, Liu S, Story M. Association between obesity and puberty timing: a systematic review and meta-analysis. Int J Environ Res Public Health 2017;14(10). 10.3390/ijerph14101266 PMC5664767 29064384

[29] Mascarenhas LP, Leite N, Titski AC, Brito LM, Boguszewski MC (2015). Variability of lipid and lipoprotein concentrations during puberty in Brazilian boys. J Pediatr Endocrinol Metab.

[30] Wang H, Peng DQ (2011). New insights into the mechanism of low high-density lipoprotein cholesterol in obesity. Lipids Health Dis.

[31] Kirkland RT, Keenan BS, Probstfield JL, Patsch W, Lin TL, Clayton GW (1987). Clayton GW, et al. Decrease in plasma high-density lipoprotein cholesterol levels at puberty in boys with delayed adolescence. Correlation with plasma testosterone levels. JAMA.

[32] Buitrago-Lopez A, van den Hooven EH, Rueda-Clausen CF, Serrano N, Ruiz AJ, Pereira MA (2015). Socioeconomic status is positively associated with measures of adiposity and insulin resistance, but inversely associated with dyslipidaemia in Colombian children. J Epidemiol Community Health.

